# Caspase-8 and BID Caught in the Act with Cardiolipin: A New Platform to Provide Mitochondria with Microdomains of Apoptotic Signals

**DOI:** 10.3390/cells14211678

**Published:** 2025-10-27

**Authors:** Patrice X. Petit

**Affiliations:** National Center for Scientific Research, CNRS UMR 8003, Paris City University, Saint-Pères, Paris Institute for Neuroscience (SSPIN), Team “Mitochondria, Apoptosis and Autophagy Signalling”, Campus Saint-Germain, 75006 Paris, France; patrice.petit@inserm.fr

**Keywords:** BID, BH3 interacting domain death agonist (also BID-FL), Cell death, CLOOH, cardiolipin peroxidized, DISC, death inducing signalling complex, GUV, giant unilamellar-vesicles, Mitochondria, MTCH2, Mitochondrial Carrier Homolog 2, OMM outer mitochondrial membrane, tBID (truncated BID at the n terminal end; p15)

## Abstract

Mitochondria play a central role in cellular bioenergetics. They contribute significantly to ATP production, which is essential for maintaining cells. They are also key mediators of various types of cell death, including apoptosis, necroptosis, and ferroptosis. Additionally, they are one of the main regulators of autophagy. This brief review focuses on BID, a molecule of the BCL-2 family that is often overlooked. The importance of the cardiolipin/caspase-8/BID-FL platform, which is located on the surface of the outer mitochondrial membrane and generates tBID, will be emphasized. tBID is responsible for BAX/BAK delocalization and oligomerization, as well as the transmission of death signals. New insights into the regulation of caspase-8 and BID have emerged, and this review will highlight their originality in the context of activation and function. The focus will be on results from biophysical studies of artificial membranes, such as lipid-supported monolayers and giant unilamellar vesicles containing cardiolipin. We will present the destabilization of mitochondrial bioenergetics caused by the insertion of tBID at the mitochondrial contact site, as well as the marginal but additive role of the MTCH2 protein, not forgetting the new players.

## 1. Introduction

Recent studies have revealed that the BCL-2 family plays a regulatory role in maintaining mitochondrial homeostasis during apoptosis. Pro-apoptotic members, including BAX, BAK, BID and Bim, promote the release of death-inducing proteins, such as cytochrome *c* [1,2], smac [3], and endonuclease G [4,5,6], from mitochondria while anti-apoptotic members, such as BCL-2 and BCL-XL, inhibit this release. Following release into the cytosol, these death-inducing proteins promote apoptotic cell destruction through multiple pathways including caspase activation and nuclear DNA fragmentation.

The BCL-2 family also alters the function of mitochondria undergoing apoptosis. Dysfunction of voltage-dependent anion channel opening, ADP/ATP exchange, the electron transport chain, oxidative phosphorylation, and calcium buffering through the action of this family of proteins have been reported [7,8,9]. While early defects in the electron transport chain can be reversed by addition of exogenous cytochrome *c*, the damage eventually becomes refractory to cytochrome *c* addition [9]. This finding suggests that pro-death BCL-2 proteins can damage mitochondrial function independently from cytochrome *c* loss.

**Figure 1:**
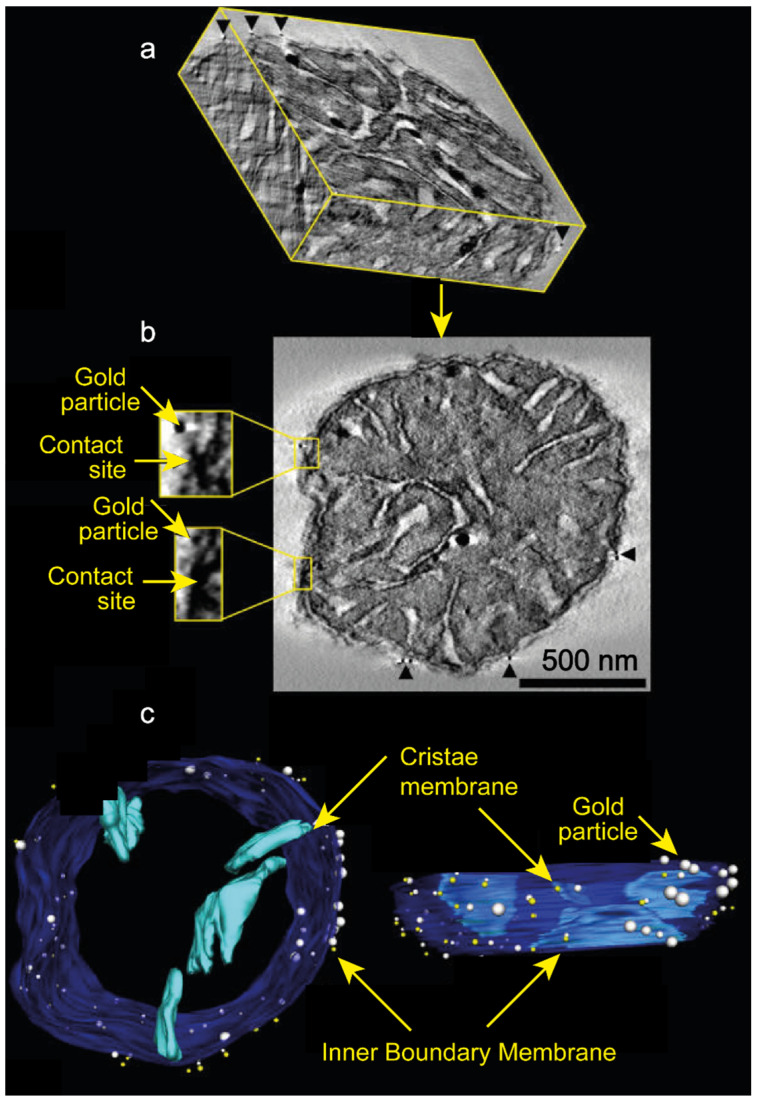
Elements of a tomographic reconstruction of a tBID_G94E_-labeled mitochondrion highlight the association of tBID with contact sites. (**a**) Portion of the tomographic volume sliced in three perpendicular planes. Thick sections were examined by intermediate voltage electron microscopy and were used to generate reconstructions of the immunogold-labeled outer membrane. Three-dimensional perspectives allowed us to track features, such as gold particles (arrowheads), along perpendicular faces. (**b**) Slice through the volume. Several gold particles are visible (arrowheads) in this 2 nm slice, including two closely associated with contact sites (insets; 3× magnification) The scale bar = 500 nm and applies to all panels. (**c**) Perpendicular views of the surface-rendered volume with selected components segmented. The visualization tool SYNU allows the surface-rendered volume to be viewed in any orientation. For clarity in visualizing the contact sites (white spheres) and gold particles (yellow spheres), the outer membrane is not shown. The inner boundary membrane is shown in dark blue and was segmented separately from individual cristae (light blue). Only four cristae are shown to demonstrate the lamellar architecture common in liver mitochondria. Where gold particles were aggregated, only one particle is shown. The IBM was made translucent in order to visualize the cristae. This is fully inspired from Lutter et al. [10], copyright © 2001 Lutter et al.; licensee BioMed Central Ltd. This is an Open Access article: verbatim copying and redistribution of this article are permitted in all media for any purpose, provided this notice is preserved.

Despite the pioneering work of Lutter et al. [10], who demonstrated that targeting the pro-apoptotic protein tBID to mitochondria depends on the presence of the mitochondria-specific lipid cardiolipin (CL) [10,11] within a potentially unique structure [12,13]. This hypothesis has not attracted the attention of researchers. CL has a defined distribution pattern within mitochondria. It is found in high concentrations throughout the inner membrane, including at contact sites where the inner and outer membranes interact. It is present at much lower concentrations elsewhere in the outer membrane, but is highly concentrated at contact sites. This suggests that tBID may localize to contact sites due to cardiolipin’s arrangement there [10] (Figure 1). The requirement for a specific cardiolipin structure that is likely defined by the contact site is consistent with the observation that no gold particles were found in regions stripped of the outer membrane exposing the cardiolipin certainly issued from the inner membrane [10].

Soon after, the ability of tBID to transfer lipids has been demonstrated in vitro [14]. Until now here the lipid transfer activity has not been measured in vivo. Additionally, the pro-oxidized form of CL, i.e., monolysocardiolipin (MLCL), has also been shown to bind tBID [15], as CL does.

Some time passed before the idea of the possible existence of an activation platform located on the outer mitochondrial membrane resurfaced [16]. The main work indeed stemmed from research on caspase-8, which has led to the new view that active caspase-8 only reaches its full activation potential when bound to cardiolipin. [17]. Admittedly, the analogy between a hypothetical platform and lipid rafts did not help to popularize this idea. In fact, the term “raft” should only be used to describe specific regions of the plasma membrane. Nevertheless, this has reinforced the concept of “platform” or hub activation at the mitochondrial outer membrane surface [18].

Other studies have identified the key role of cardiolipin (CL) in the mitochondrial membrane [19]. These studies essentially concerned the relationship between cardiolipin and cytochrome *c* in the intermembrane space [20,21,22,23]. However, that is not the focus of the present review, which centers on the concept of protein-lipid interaction. In our case, we focus on the interaction of pre-activated caspase-8 at the DISC level and its recruitment by cardiolipin to reach full activation at the surface of the mitochondrial outer membrane [19]. It then interacts with BID to generate tBID, which greatly disturbs mitochondrial bioenergetic homeostasis and recruits the pore-forming molecules BAX and/or BAK leading to mitochondrial permeability completion and cytochrome *c* release. The marginal work of the protein MTCH2 is also envisaged as well as the participation of new BH-3 interacting molecules.

## 2. The Activation Platform: Cardiolipin, Caspase-8, and BID

The title of this comment is inspired voluntary from the review article by Kantari Ch. and Walczak H. [24], since it has recognized for the first time the importance of a platform of activation located at the mitochondrial membrane.

As recently explained, the activation platform is at work in the multiple entanglements of different cell death pathways, which justifies a precise definition and understanding of the “caspase-8/cardiolipin/BID” complex. This short review highlights the precise manner in which this protein associates with lipids in various cell death signaling pathways, as well as its more recently discovered role in autophagy. It was necessary to emphasize the importance of this unique complex at the mitochondrial membrane and distinguish its action from that of MTCH2 [25] or other partners such as ATR [26] and ATM [27], which also possess a BH3-like binding domain.

A better understanding of the role of “caspase-8/cardiolipin/BID” has also recently been useful in understanding Barth syndrome, where mutations in the TAFAZIN gene lead to cardiolipin abnormalities that affect both platform formation at the outer mitochondrial surface, apoptosis [28] and autophagy signal transduction [29].

This is a short review and a comment and discussion of the recent publication entitled “BID Protein: A Participant in the Apoptotic Network with Roles in Viral Infections” by Wyżewski Z. et al. [30], These authors minimize the role of the activation platform formed by cardiolipin/Caspase-8/Bid in favor of the MCTH2 protein, but it is likely to be the other way around. MTCH2 acts as a facilitator of BID recruitment to the outer mitochondrial surface facing the cytoplasm, but the main actors are indeed the CL/Caspase-8-activated that recruited BID for cleavage.

### 2.1. Cardiolipin as a Key Mitochondrial Determinant

Mitochondria are organelles of endosymbiotic origin, surrounded by two membranes, the outer and the inner, which define a very narrow intermembrane space. Because of their endosymbiotic origin, mitochondria retain bacterial characteristics [31]. This selective disruption of the two-billion-year-old endosymbiotic relationship enables mitochondria to function as intracellular signaling hubs. These signals include proteins, nucleic acids, phospholipids, metabolites, and reactive oxygen species, which are released in many ways from the mitochondria and/or land on the mitochondrial membrane surface. Despite its almost exclusive location in the inner mitochondrial membrane [32,33,34], there is a certain amount of CL located at the outer mitochondrial membrane, at the contact sites, in normal conditions. Under apoptotic stimuli, CL redistributes to the outer mitochondrial membrane (OMM) and provides a unique lipid microenvironment mostly, but not uniquely, located at the so called “mitochondrial contact site and cristae organizing system” (MICOS) [35,36]. Its unusual dimeric structure (four acyl chains) confers not only a non-bilayer conformation but also creates specific docking sites for proteins that carry out the early steps of the apoptotic cascade [19].

In this context, following the activation of a death receptor, the death-induced signaling complex (DISC) assembles within specialized platforms of the plasma membrane of the cell. These domains are called lipid rafts [37,38]. Glycosphingolipid molecules, including gangliosides, are concentrated in these domains [39]. It is also complexed with several glycoproteins involved in signal transduction pathways. These include tyrosine kinase receptors, mono- or heterotrimeric G proteins, glycosyl phosphatidylinositol-anchored proteins, Src-like tyrosine kinases, and protein kinase C isozymes [40]. Proenzymes of the apical caspases, mainly pro-caspase-8 are recruited [41,42] and undergo activation by proximity. When enough DISCs are formed, active apical caspases directly process and turn effector caspases on.

Alternatively, in type II cells, additional amplificatory mechanisms are required [42,43]. In these cells, caspase-8 cleaves a BH3 member of the BCL-2 family of proteins, BID, triggering its translocation to mitochondria leading to cytochrome *c* release [42]. In CEM lymphoblastoid T cells (3D-culture), caspase-8, as well as tBID and BAX, are only recruited to the outer mitochondrial membrane fraction, upon CD95/Fas ligation [44]. BAK forms oligomers with BAX on the outer mitochondrial membrane and is found in the proposed CL-enriched microdomains. BAK is also present in BAX-negative primary cells, such as hepatocytes.

In several in vitro studies using reconstituted membrane systems (e.g., proteo-liposomes containing defined lipid compositions) [45,46,47,48], CL has been shown to directly target caspase-8 on the mitochondrial surface [17]. Caspase-8 is pre-activated at the receptor level (plasma membrane), but only becomes fully activated when it binds to CL at the mitochondrial outer membrane [17,49]. This localization is critical because caspase-8 then recruits and cleaves BID, to its truncated form (tBID), which is highly competent to permeabilize mitochondria alone [50] or with BAX [48,51] and BAK [52,53,54]. It is now clear that CL acts as a critical signaling platform that orchestrates apoptosis by integrating signals from various death-inducing proteins.

### 2.2. Caspase-8, BID, tBID and Lipid Binding

#### 2.2.1. Interaction with Lipids

**Figure 2:**
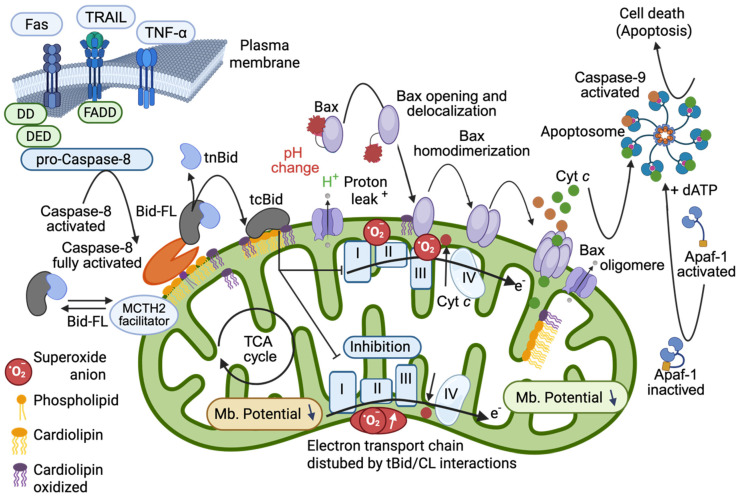
Schematic interpretation of the mitochondrial changes induced the formation of platform with Caspase-8 fully activated, cardiolipin binding of BID and tBID insertion into OMM.

Once formed under the activity of caspase-8 activated, tBID binds with high affinity to CL [16,17]. This binding is not merely passive; it induces significant remodeling of the mitochondrial membrane that can affect the stability and organization of the electron transport chain (ETC). tBID binding instantaneously prompts a change in curvature that affects the CL at the outer membrane (less than 5%) and the CL of the IMM. This dissociates the mitochondrial respiratory super-complexes [16]. tBID binding induces changes in the oxidative activity of the respiratory chain and also enhances the locally toxic superoxide anions that promote an immediate oxidation of CL. The oxidation events yield monolysocardiolipin (MLCL) as well as an increased production of phosphatidylglycerol (PG). Both MLCL and phosphatidylglycerol have been implicated in further destabilization of the electron transport chain, thereby exacerbating mitochondrial dysfunction and bioenergetic collapse. In fact, oxidized cardiolipin moved to the OMM where it enhance amount of tBID bound (Figure 2).

An informed approach to understand how tBID anchors itself to membranes revealed that two alpha helices are essential due to their charges. The alpha-helices aH6 and aH7, which form the hairpin domain of tBID, effectively mediate the insertion of BID into mitochondrial membranes. There, the BH3 domain interacts with BAX or BAK, and the alpha-helices are essential for cytochrome *c* release [55]. When using artificial monolayer, the negatively charged CL greatly enhances the insertion of aH6 peptide (EKTMLVLALLLAKKVASH) into the phospholipid monolayer. Modifying two charged amino acid residues in αH6 (creating αH6m, with two lysines at positions 157 and 158 replaced with two alanines, i.e., EKTMLVLALLLAAAVASH, to reduce its charge from +2.5 to +0.5) abolished its insertion and in vivo effects [55]. Curiously and surprisingly also, the electro-permeabilization of such aH6 peptide alone (at mM concentration) is able to promote a mitochondrial membrane permeabilization that ended in the death of cells. Accompanied at the bioenergetic level by all the stigmata obtained with tBID (in the nM range), i.e., ADP-stimulated respiration is inhibited (State-3) and state-4 respiration is slightly uncoupled, the mitochondrial DYm drops and mitochondria swell. As an alternative to the use of purified peptides, diverse constructs have been expressed in cells [56] and the results validated the importance of the two alpha helices for tBID insertion into mitochondrial membrane and of the presence of a BH3-domain for a linkage to BAX [57,58] or BAK [52].

Many of these conclusions are based on experiments using either isolated mitochondria [16], artificial membranes [16], liposomes [47,59,60] or even giant unilamellar systems [45,46] with variable CL content to reconstitute apoptotic signaling in vitro. In such systems, the addition of activated caspase-8 leads to BID cleavage and subsequent incorporation of tBID into membranes, with biochemical analyses demonstrating tBID oligomerization, membrane permeabilization and disruption of mitochondrial bioenergetics. These aspects will be developed below. It is clear that soluble tBID progresses through different states, i.e., from a loosely bound state to a superficially inserted state to a potent membrane-inserted state where it undergoes an oligomerization process similar to that observed for BAX [19].

Refined model of the pro-apoptotic function of tBID that sets the importance of tBID/CL interactions. First, BID-FL binds to the CL present at the contact site (or MICOS) via its charged α-helices and destabilizes the mitochondrial membrane. The interaction of tBID with CL (i.e., following the interaction of preactivated caspase-8 with CL) disrupts the organization of the mitochondrial membrane at the contact sites (and certainly throughout the entire IMM). The induced structural changes, as well as the immediate generation of peroxidized CL, disrupt the activity of the electron transport chain complexes (reduced electron transport rate) and lead to cytosolic acidification, mitochondrial ROS production, and total mitochondrial lipid peroxidation. This environment can prime the activation of BAX and/or BAK and allow their activation and delocalization (with first homodimerization and then oligomerization at the membrane to form a pore). Moreover, tBID interacts through its BH3 domain with BAX and/or BAK and enhances membrane anchorage, which results in the liberation of cytochrome *c* to form apoptosomes in the cytoplasm with dATP, APAF-1 and caspase-9, which become activated and act as the main executioner caspases in cell death. This schematic interpretation was drawn partly with the Scientific Image (Adobe illustrator 2025) and Illustration Software (Biorender) and is partly inspired from [56] but stayed under the copyright of @Patrice X. Petit.

#### 2.2.2. Protein–Protein Interactions

As described above, the pre-activated caspase-8 at the DISC binds first to CL at the contact sites (or MICOS), acquires its fully activated form and destabilizes the mitochondrial membrane [17,19]. The interaction of BID with the protein MTCH2 facilitates its recruitment and certainly also acts as a titration process (since the BID protein gives rise to tBID, acting as a very efficient protein in the nM range). The generated tBID, strongly interacting with CL, disrupts the organization of the mitochondrial membrane at the contact sites. It then induces structural changes in the membrane, as well as the immediate generation of peroxidized CL. This disrupts the activity of the electron transport chain complexes, reducing the electron transport rate. It also leads to cytosolic acidification, pH changes at the vicinity of the OMM, mitochondrial ROS production, and mitochondrial lipid peroxidation [16] (Figure 2). This environment (pH change and superoxide anion within the membrane) [61] can prime the activation of BAX and/or BAK and allow their activation and delocalization (with first a homodimerization and then an oligomerization at the membrane to form a pore).

#### 2.2.3. Regulatory Cross-Talk

At the same time, through its BH3 domain, tBID interacts with BAX and/or BAK and enhances membrane anchoring, resulting in the release of cytochrome *c* to form the apoptosome in the cytoplasm with dATP, APAF-1 and caspase-9, which are activated and act as the main executioner caspases in cell death.

## 3. MTCH2 at the Mitochondrial Membrane

### 3.1. MTCH2 as a Facilitator, Not an Enzyme!

MTCH2 (Mitochondrial Carrier Homolog 2) has been identified as a mitochondrial protein that interacts with tBID at the outer mitochondrial membrane surface [25]. Although its nomenclature might suggest a potential transport or catalytic role as a “carrier,” current evidence does not support any intrinsic enzymatic activity associated with MTCH2. Indeed, structural and biochemical analyses of MTCH2 have not revealed any domains that are consistent with known catalytic activities. Rather, MTCH2 plays a regulatory role in apoptosis by modulating the spatial localization and/or the kinetics of tBID’s engagement with the OMM. Its function appears to be primarily structural or as an adaptor protein. Very recently, MTCH2 has been described as an insertase [62] that mediated the insertion of diverse tail-anchored, signal-anchored [62] or/and scramblase [63] (even if this activity has not been tested “in vivo”), and multi-pass proteins (α-helical proteins). Recent studies have revealed the plethora of physiological and pathological functions of MTCH2 in metabolic diseases, neurodegenerative diseases, cancers, embryonic development, and reproduction. These functions include regulating mitochondrial apoptosis, shifting metabolism between glycolysis and oxidative phosphorylation, and controlling mitochondrial fusion/fission and epithelial–mesenchymal transition. These functions are far beyond its unique docking capabilities [64].

### 3.2. Does MTCH2 Interfere with the tBID-Cardiolipin System?

Importantly, experimental data obtained from “in vitro” systems have demonstrated that the presence of MTCH2 does not obstruct tBID from directly binding to CL [65]. For example, in reconstituted systems in which proteo-liposomes mimicked the mitochondrial outer membrane—both in the absence and presence of MTCH2—tBID showed a strong binding preference for CL. This observation suggests that while MTCH2 may serve as a docking partner that accelerates or stabilizes the recruitment of tBID to mitochondria, it does not replace or competitively inhibit tBID inherent affinity for CL. As a result, even in cells with intact MTCH2, tBID retains its ability to bind to CL or other lipids such as MLCL and phosphatidylglycerol, ultimately leading to disturbances in mitochondrial bioenergetic homeostasis through mitochondrial membrane structures and the electron transport chain.

### 3.3. Redundant Functions? Or a More Complex Situation

CL-deficient cells were viable in the presence of glucose but showed impaired oxidative phosphorylation and an inability to grow in galactose. In these cells, CL did not appear to be necessary for tBID-induced BAX activation or apoptosis in response to TRAIL treatment [20]. However, the fact that tBID also has a marked affinity for both monolysocardiolipin MLCL and PG, which is highly enriched in these cells, was forgotten, and so the interpretation is clear—tBID can bind other lipids, either the MLCL (first CL derivative in an oxidative context) or PG (providing a binding alternative even though tBID has a lower affinity for PG). This may explain why tBID still exerts its function.

Conversely, downregulation of MTCH2 failed to prevent the recruitment of tBID to mitochondria under apoptotic conditions [65]. However, when both CL and MTCH2 were depleted, a significant reduction in tBID recruitment was observed. This suggests that CL and MTCH2 may have redundant docking functions. Even when CL drives caspase-8 cleavage activity, MTCH2 makes the difference!

## 4. Some New Players Enter the Game Together with tBID

The phosphatidylinositol 3-kinase (PI3K)-like protein kinase ATR (*Ataxia telangiectasia* and Rad3-related) is central to the maintenance of genome integrity in the context of DNA damage [66,67,68] and is also a key protein that prevents the onset of cancer [69]. ATR is a checkpoint kinase that phosphorylates hundreds of downstream proteins during DNA damage responses [70]. ATR acts as a complex with the ATR-interacting protein (ATRIP). This complex senses replicative stress-induced DNA damage, activates checkpoints, arrests the cell cycle, and facilitates repair to restore DNA integrity [71].

**Figure 3:**
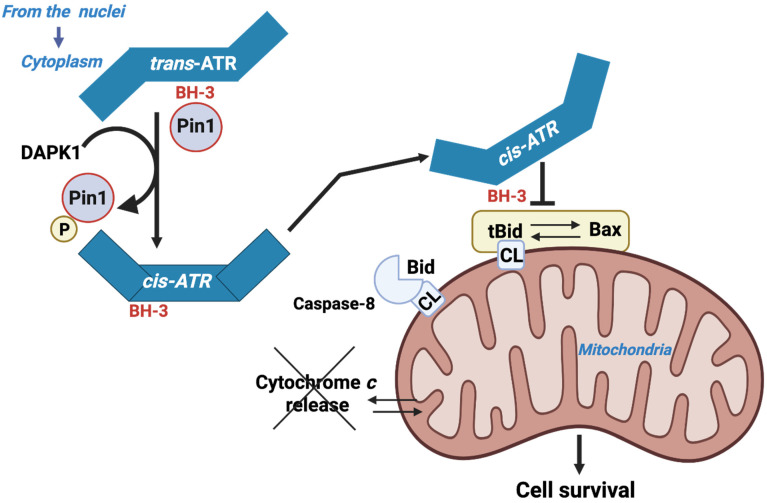
ATR as a BH3 only domain that interacts with tBID.

ATR has a BH3-like domain allowing it to interact with the tBID counterpart of BID. ATR then exerts an antiapoptotic effect similar to that of BCL-2 and BCL-XL [26]. This activity is patent in the context of UV-induced apoptosis (Figure 3). The action of ATR appears to be independent of its hallmark checkpoint and kinase activities and does not involve its usual partner, ATRIP. ATR’s antiapoptotic action is mediated by its interaction with tBID at the mitochondrial membrane. This interaction blocks cytochrome *c* release from the intermembrane space and subsequent apoptosis. Pin1 (peptidylprolyl cis/trans isomerase NIMA-interacting) downregulates the mitochondrial localization of ATR by isomerizing it from the cis- to trans-isomere at the phosphorylated Ser428-Pro429 motif. UV exposure inactivates Pin1 via Death-Associated Protein Kinase 1 (DAPK1), which belongs to a family of five serine/threonine kinases that possess tumor-suppressive functions and mediate a wide range of cellular processes, including apoptosis and autophagy. DAPK1 stabilizes the pro-survival cis-isomeric ATR [26,72].

### 4.1. The ATR Protein Kinase Is a New Player in the Game

Localization of ATR to mitochondria may occur either through the binding of ATR-H to mitochondria-bound tBID or through cytoplasmic ATR-H-tBID interactions to facilitate mitochondrial localization of the ATR-H-tBID complex [26]. More precisely, ATR-H (cis-ATR) formation occurs in the cytoplasm due to Pin1 inactivation via UV-induced phosphorylation at Ser71 by DAPK1, or due to genetic deficiency. ATR-H can localize to the mitochondria, where it interacts with tBID that has been inserted into the outer mitochondrial membrane via its now accessible BH3-like domain. Alternatively, ATR-H can bind to cytosolic tBID prior to localization to the mitochondria. The result is that ATR-H functions as an antiapoptotic protein, preventing the further recruitment of BAX to the mitochondria, subsequent BAX/BAK activation, and cytochrome *c* release, thus deterring apoptosis. The unexpected cytoplasmic response of ATR is undoubtedly the basis for the observed antiapoptotic role of ATR in suppressing carcinogenesis and its inhibitory action in sensitizing anticancer agents to kill cancer cells. This information underscores the pivotal role of tBID in determining cell fate between apoptosis and carcinogenesis.

### 4.2. ATM Kinase Is Also a Player in the Game Together with tBID

Several studies have reported that BID is phosphorylated by the protein kinase ataxia telangiectasia mutated (ATM) and that BID plays a protective role during DNA damage [27,73,74,75]. There is some controversy regarding in vitro studies [76]; however, the fact that non-phosphorylated BID-S61A/S78A-knock-in mice are more sensitive to IR-induced death [74] constitutes evidence that BID, via its activities in the mitochondria, is a pivotal regulator of the DNA damage response in vivo.

### 4.3. MTCH2

#### 4.3.1. MTCH2 as a Facilitator

While MTCH2 acts as a facilitator of tBID recruitment to mitochondria, this is not its only or main function. Numerous publications have demonstrated that MTCH2 plays a pivotal role in regulating both lipid metabolism and mitochondrial dynamics [77,78,79,80,81,82,83]. Additionally, several studies have reported that BID plays a role in regulating obesity/metabolism [82,84,85]. Based on these and other studies on CL and respiration mentioned in this review, it is tempting to speculate that BID regulates metabolism, obesity and respiration by interacting with and regulating MTCH2 activity. Furthermore, these studies suggest that BID/MTCH2 may regulate metabolism and be involved in mitochondrial outer membrane permeabilization (MOMP). MTCH2 has been reported to function as both an MOM insertase [62,63] and a phospholipid scramblase [62,63]. VDAC also functions as a phospholipid scramblase [63,83]. Upon reconstitution into membrane vesicles, dimers of human VDAC1 and VDAC2 rapidly translocate phospholipids across the bilayer via a mechanism that is independent of their channel activity. Therefore, it is reasonable to speculate that VDAC isoforms, which are members of a superfamily of beta-barrel proteins, act as a distinct class of phospholipid scramblases in vivo, separate from the alpha-helical scramblase proteins that import lipids into mitochondria. BID has been described as a potential lipid transfer protein [86]. It is also possible that this activity involves cooperation with VDAC and/or MTCH2. The regulation of lipid composition and protein insertion into the mitochondrial outer membrane (MOM) may be involved in the process of mitochondrial outer membrane permeabilization (MOMP).

#### 4.3.2. New Functions of MTCH2, Aside from BID Signaling

MTCH2 plays a key role in regulating adipocyte differentiation and lipid homeostasis [23,27,28,29,30,31,32]. Genetic alterations in MTCH2 have been linked to various disease phenotypes, including obesity, Alzheimer’s disease and cancer [64]. Notably, genome-wide association studies have identified MTCH2 variants associated with an increased risk of obesity and diabetes [34]. MTCH2 has been recently demonstrated to be able to set the influx of fatty acids (FAs) into mitochondria via changing the sensing of malonyl-CoA by the carnitine palmitoyltransferase 1 (CPT1), thereby influencing both mitochondrial function and lipid metabolism [87].

Interestingly, recent outcomes placed MTCH2 as a key regulator of melanoma proliferation. MTCH2 appears to enhance the expression and nuclear translocation of NRF2, which in turn increases RRM1 expression and promotes melanoma cell proliferation. These recent findings elucidate a mechanistic link between MTCH2 and the NRF2-RRM1 axis in melanoma proliferation, highlighting potential therapeutic targets for intervention [88].

### 4.4. Humanin as an Inhibitor That Sequesters tBID into Fiber Structures

A wide class of mitochondrial retrograde signaling peptides known as mitochondrial-derived peptides (MDPs) has been described for its ability to interact with endogenous BCL-2 proteins. All of these peptides affect MOMP-mediated apoptosis [89,90]. Humanin (HN) is a natural short peptide that protects cells against various stress conditions and apoptosis. More precisely, humanin is a mitochondrial-derived peptide expressed from an alternative open reading frame (ORF) in the mitochondrial genome. It inhibits apoptosis by interacting with pro-apoptotic BCL-2 proteins, such as tBID or BAX. Recently, it was reported that HN-mediated BAX sequestration and inactivation result in the formation of β-sheet fibers in vitro. [91]. With regard to BID, HN also appears to initiate the formation of fibers. The use of biophysical approaches involving spectroscopic techniques and mass analysis, in conjunction with protein fragmentation and electron microscopy, enables the formation of fiber structures to be validated when HN and BID interact. Moreover, β-sheet structures and fibrillation can be observed [92]. The fibers have a uniform diameter, with BID situated in the fiber core. Light scattering experiments under various conditions illustrate the sensitivity of fiber formation to environmental conditions (i.e., the vicinity of the mitochondrial outer membrane). This suggests that the interaction between BID and HN, which promotes fiber formation, may be modulated by intracellular pH, temperature, and membrane localization. Since HN can also interact with MCL-1, peptides in this family are being investigated intensively in the hope that targeting them will lead to positive results for therapeutic purposes.

## 5. In Vitro Systems Demonstrating the Role of Cardiolipin

### 5.1. Proteoliposome Models and Giant Unilamellar Vesicles

One of the most convincing sets of experiments has been performed using proteoliposome systems, where defined mixtures of lipids (including CL, phosphatidylglycerol, and MLCL) recreate key aspects of the mitochondrial outer membrane. In these systems, addition of recombinant caspase-8 and BID leads to the formation of tBID and its subsequent insertion into the membranes. The high binding affinity of tBID for CL and oxidized CL derivatives (MLCL) is confirmed by changes in membrane permeability.

A new technique allowing the analysis of giant unilamellar vesicles in flow [45,93] has shown that tBID binds to GUVs in the presence of CL [15,16]. Moreover, tBID induces changes in the membrane curvature of GUVs with sufficient strength to disrupt the vesicles that resealed at a smaller size [46].

### 5.2. Purified Mouse Mitochondria

A mixture of recombinant active Caspase-8 + BID recombinant or recombinant tBID induces an instantaneous destabilization of mitochondrial bioenergetic homeostasis “in vitro” [16]. tBID action results in a mild uncoupling of mitochondrial state-4 respiration, associated with an inhibition of adenosine diphosphate (ADP)-stimulated respiration (State-3) and phosphorylation rate. All these events being inhibited in mitochondria overexpressing BCL-2 and BCL-XL that are purified from transgenic BCL-2 [94] or BCL-XL [95] mice. The inhibition of state 3 respiration is mediated by the reorganization of CL within mitochondrial membranes, which indirectly affects the activity of the ADP/ATP translocator (See Figure 1).

### 5.3. Yeast Mitochondria

CL-deficient yeast mitochondria [from crd1Δ yeast cells [37]] showed no respiratory inhibition by tBID, demonstrating the absolute requirement of CL for tBID binding and activity. In contrast, wild-type yeast mitochondria exhibited inhibition of ADP-stimulated respiration similar to that seen in mammalian cells, which was associated with reduced ATP synthesis. These events suggest that mitochondrial lipids, rather than proteins, are the key determinants of tBID-induced destabilization of mitochondrial bioenergetics [38]. It should be noted that the MTCH2 protein is absent in yeast cells [39].

### 5.4. Biochemical and Biophysical Approach “In Vitro”

Other approaches include surface plasmon resonance (SPR) [44] and fluorescence resonance energy transfer (FRET) [45] assays, which help to quantify the binding kinetics between tBID and membranes of different lipid compositions, but also its relationship with BAX and BAK. They support the key role of the tBID BH3 domain in the interaction with BAX or BAK. A point that is rarely discussed is that once tBID binds to CL, and induces mitochondrial membrane changes and a bioenergetic defect, there is a local change in pH at the vicinity of the OMM [16] that works as a key event in the delocalization of BAX to the membrane and in its further oligomerization to form pore into the mitochondrial membrane [61]. These experiments have underscored the specificity and strength of the tBID-CL interaction and further support the conclusion that modifications to CL via oxidative processes linked to apoptotic signaling (such as oxidation to MLCL) enhance the ability of tBID to disrupt mitochondrial function and then carry cells to a point of no return.

## 6. Conclusions

CL, caspase-8, and BID form an essential activation platform [16,17,96,97] on the mitochondrial outer membrane where CL acts both as a terminal activator of caspase-8 (resulting in a full activation of caspase-8 that has already been preactivated) and as a receptor for tBID [16,17]. The recruitment of tBID to CL-rich domains leads to critical alterations in the electron transport chain, contributing to mitochondrial dysfunction and the initiation of apoptosis [16].

MTCH2 functions as a facilitator for tBID recruitment without compromising tBID’s ability to directly bind CL. Despite its involvement in apoptotic signaling, MTCH2 has not been shown to carry enzymatic activity; its role is regulatory and structural rather than catalytic.

Reconstituted systems using proteoliposomes and other biophysical assays have convincingly demonstrated the key role of CL in targeting caspase-8 to the mitochondrial membrane, mediating BID cleavage, and promoting the membrane integration of tBID. Importantly, these systems clarify that the presence of MTCH2 does not impede tBID’s direct interactions with CL. A conclusion that underlines the independence of tBID lipid-binding activity from MTCH2.

Overall, the convergence of these findings offers a robust model wherein CL acts as a critical signaling hub during apoptosis, and MTCH2, while important for the efficient recruitment of tBID, does not alter the fundamental biochemical events driven by tBID’s CL binding.

## Data Availability

No new data were created or analyzed in this study. Data sharing is not applicable to this article.

## Abbreviations

ATM, protein kinase mutated from ataxia telangiectasia; ATR, Ataxia telangiectasia and Rad3-related protein; BAK, BCL-2 antagonist/killer 1; BAX, apoptosis regulator BAX, also known as bcl-2-like protein 4; BCL-2, derived from B-cell lymphoma 2, is the founding member of an important family of pro- or antiapoptotic proteins; BCL-XL, B-cell lymphoma extra-large is one of the anti-apoptotic proteins of the Bcl-2 family; BID, BH3 interacting-domain death agonist; CL, cardiolipin; CPT1, Carnithine palmitoyltransferase; DISC, death-inducing signalling complex; tBID, its truncated form at the n-terminus, p15; CD95/Fas (also known as APO-1 or CD95) belongs to the subgroup of the tumor necrosis factor receptor (TNF-R) family that contains an intracellular “death domain”; GUV, giant unilamellar vesicles; IMM, inner mitochondrial membrane; MICOS, also known as the mitochondrial contact site and cristae organizing system; MLCL, Monolysocardiolipin; MOMP, mitochondrial outer membrane permeability; OMM, outer mitochondrial membrane; Smac/Diablo, Second Mitochondria-derived Activator of Caspases and DIABLO, Direct IAP-Binding protein with Low PI proteins; MTCH2, Mitochondrial carrier homolog 2; VDAC, voltage anionic channel; and XIAP, X-linked inhibitor of apoptosis protein.

## References

[1] Li H., Zhu H., Xu C.J., Yuan J. (1998). Cleavage of BID by caspase 8 mediates the mitochondrial damage in the Fas pathway of apoptosis. Cell.

[2] Luo X., Budihardjo I., Zou H., Slaughter C., Wang X. (1998). Bid, a bcl-2 interacting protein, mediates cytochrome *c* release from mitochondria in response to activation of cell surface receptors. Cell.

[3] Du C., Fang M., Li Y., Li L., Wang X. (2000). Smac, a mitochondrial protein that promotes cytochrome c-dependent caspase activation by eliminating IAP inhibition. Cell.

[4] Li L.Y., Luo X., Wang X. (2001). Endonuclease G is an apoptotic DNase when released from mitochondria. Nature.

[5] Parrish J., Li L., Klotz K., Ledwich D., Wang X., Xue D. (2001). Mitochondrial endonuclease G is important for apoptosis in *C. elegans*. Nature.

[6] Widlak P., Li L.Y., Wang X., Garrard W.T. (2001). Action of recombinant human apoptotic endonuclease G on naked DNA and chromatin substrates: Cooperation with exonuclease and DNase I. J. Biol. Chem..

[7] Vander Heiden M.G., Li X.X., Gottleib E., Hill R.B., Thompson C.B., Colombini M. (2001). Bcl-xL promotes the open configuration of the voltage-dependent anion channel and metabolite passage through the outer mitochondrial membrane. J. Biol. Chem..

[8] Vander Heiden M.G., Chandel N.S., Schumacker P.T., Thompson C.B. (1999). Bcl-xL prevents cell death following growth factor withdrawal by facilitating mitochondrial ATP/ADP exchange. Mol. Cell.

[9] Mootha V.K., Wei M.C., Buttle K.F., Scorrano L., Panoutsakopoulou V., Mannella C.A., Korsmeyer S.J. (2001). A reversible component of mitochondrial respiratory dysfunction in apoptosis can be rescued by exogenous cytochrome c. EMBO J..

[10] Lutter M., Perkins G.A., Wang X. (2001). The pro-apoptotic Bcl-2 family member tBid localizes to mitochondrial contact sites. BMC Cell Biol..

[11] Lutter M., Fang M., Luo X., Nishijima M., Xie X., Wang X. (2000). Cardiolipin provides specificity for targeting of tBid to mitochondria. Nat. Cell Biol..

[12] Ardail D., Lerme F., Louisot P. (1990). Further characterization of mitochondrial contact sites: Effect of short-chain alcohols on membrane fluidity and activity. Biochem. Biophys. Res. Commun..

[13] Ardail D., Privat J.P., Egret-Charlier M., Levrat C., Lerme F., Louisot P. (1990). Mitochondrial contact sites. Lipid composition and dynamics. J. Biol. Chem..

[14] Esposti M.D., Erler J.T., Hickman J.A., Dive C. (2001). Bid, a widely expressed proapoptotic protein of the Bcl-2 family, displays lipid transfer activity. Mol. Cell Biol..

[15] Esposti M.D., Cristea I.M., Gaskell S.J., Nakao Y., Dive C. (2003). Proapoptotic Bid binds to monolysocardiolipin, a new molecular connection between mitochondrial membranes and cell death. Cell Death Differ..

[16] Gonzalvez F., Pariselli F., Dupaigne P., Budihardjo I., Lutter M., Antonsson B., Diolez P., Manon S., Martinou J.C., Goubern M. (2005). tBid interaction with cardiolipin primarily orchestrates mitochondrial dysfunctions and subsequently activates Bax and Bak. Cell Death Differ..

[17] Gonzalvez F., Schug Z.T., Houtkooper R.H., MacKenzie E.D., Brooks D.G., Wanders R.J., Petit P.X., Vaz F.M., Gottlieb E. (2008). Cardiolipin provides an essential activating platform for caspase-8 on mitochondria. J. Cell Biol..

[18] Sorice M., Manganelli V., Matarrese P., Tinari A., Misasi R., Malorni W., Garofalo T. (2009). Cardiolipin-enriched raft-like microdomains are essential activating platforms for apoptotic signals on mitochondria. FEBS Lett..

[19] Gonzalvez F., Gottlieb E. (2007). Cardiolipin: Setting the beat of apoptosis. Apoptosis.

[20] Vladimirov Y.A., Proskurnina E.V., Izmailov D.Y., Novikov A.A., Brusnichkin A.V., Osipov A.N., Kagan V.E. (2006). Mechanism of activation of cytochrome C peroxidase activity by cardiolipin. Biochemistry.

[21] Mohammadyani D., Yanamala N., Samhan-Arias A.K., Kapralov A.A., Stepanov G., Nuar N., Planas-Iglesias J., Sanghera N., Kagan V.E., Klein-Seetharaman J. (2018). Structural characterization of cardiolipin-driven activation of cytochrome c into a peroxidase and membrane perturbation. Biochim. Biophys. Acta Biomembr..

[22] Li M., Mandal A., Tyurin V.A., DeLucia M., Ahn J., Kagan V.E., van der Wel P.C.A. (2019). Surface-Binding to Cardiolipin Nanodomains Triggers Cytochrome c Pro-apoptotic Peroxidase Activity via Localized Dynamics. Structure.

[23] Kagan V.E., Bayir H.A., Belikova N.A., Kapralov O., Tyurina Y.Y., Tyurin V.A., Jiang J., Stoyanovsky D.A., Wipf P., Kochanek P.M. (2009). Cytochrome c/cardiolipin relations in mitochondria: A kiss of death. Free Radic. Biol. Med..

[24] Kantari C., Walczak H. (2011). Caspase-8 and Bid: Caught in the act between death receptors and mitochondria. Biochim. Biophys. Acta.

[25] Zaltsman Y., Shachnai L., Yivgi-Ohana N., Schwarz M., Maryanovich M., Houtkooper R.H., Vaz F.M., De Leonardis F., Fiermonte G., Palmieri F. (2010). MTCH2/MIMP is a major facilitator of tBID recruitment to mitochondria. Nat. Cell Biol..

[26] Hilton B.A., Li Z., Musich P.R., Wang H., Cartwright B.M., Serrano M., Zhou X.Z., Lu K.P., Zou Y. (2015). ATR Plays a Direct Antiapoptotic Role at Mitochondria, which Is Regulated by Prolyl Isomerase Pin1. Mol. Cell.

[27] Kamer I., Sarig R., Zaltsman Y., Niv H., Oberkovitz G., Regev L., Haimovich G., Lerenthal Y., Marcellus R.C., Gross A. (2005). Proapoptotic BID is an ATM effector in the DNA-damage response. Cell.

[28] Gonzalvez F., D’Aurelio M., Boutant M., Moustapha A., Puech J.P., Landes T., Arnaune-Pelloquin L., Vial G., Taleux N., Slomianny C. (2013). Barth syndrome: Cellular compensation of mitochondrial dysfunction and apoptosis inhibition due to changes in cardiolipin remodeling linked to tafazzin (TAZ) gene mutation. Biochim. Biophys. Acta.

[29] Saric A., Andreau K., Armand A.S., Moller I.M., Petit P.X. (2015). Barth Syndrome: From Mitochondrial Dysfunctions Associated with Aberrant Production of Reactive Oxygen Species to Pluripotent Stem Cell Studies. Front. Genet..

[30] Wyzewski Z., Gregorczyk-Zboroch K.P., Mielcarska M.B., Switlik W., Niedzielska A. (2025). Bid Protein: A Participant in the Apoptotic Network with Roles in Viral Infections. Int. J. Mol. Sci..

[31] Murphy M.P., O’Neill L.A.J. (2024). A break in mitochondrial endosymbiosis as a basis for inflammatory diseases. Nature.

[32] Corcelli A., Schlame M. (2016). Cardiolipin as key lipid of mitochondria in health and disease. 2016, 2nd Edition, Florence, Italy, September 30–October 1, 2015. Chem. Phys. Lipids.

[33] Esposti M.D. (2002). Lipids, cardiolipin and apoptosis: A greasy licence to kill. Cell Death Differ..

[34] Fox C.A., Ryan R.O. (2022). Studies of the cardiolipin interactome. Prog. Lipid Res..

[35] Khosravi S., Harner M.E. (2020). The MICOS complex, a structural element of mitochondria with versatile functions. Biol. Chem..

[36] Benaroya H. (2024). Mitochondria and MICOS–Function and modeling. Rev. Neurosci..

[37] Simons K., Ikonen E. (1997). Functional rafts in cell membranes. Nature.

[38] Ikonen E. (2001). Roles of lipid rafts in membrane transport. Curr. Opin. Cell Biol..

[39] Hakomori S.I. (2008). Structure and function of glycosphingolipids and sphingolipids: Recollections and future trends. Biochim. Biophys. Acta.

[40] Simons K., Toomre D. (2000). Lipid rafts and signal transduction. Nat. Rev. Mol. Cell Biol..

[41] Garofalo T., Misasi R., Mattei V., Giammarioli A.M., Malorni W., Pontieri G.M., Pavan A., Sorice M. (2003). Association of the death-inducing signaling complex with microdomains after triggering through CD95/Fas. Evidence for caspase-8-ganglioside interaction in T cells. J. Biol. Chem..

[42] Scaffidi C., Fulda S., Srinivasan A., Friesen C., Li F., Tomaselli K.J., Debatin K.M., Krammer P.H., Peter M.E. (1998). Two CD95 (APO-1/Fas) signaling pathways. EMBO J..

[43] Fulda S., Scaffidi C., Pietsch T., Krammer P.H., Peter M.E., Debatin K.M. (1998). Activation of the CD95 (APO-1/Fas) pathway in drug- and gamma-irradiation-induced apoptosis of brain tumor cells. Cell Death Differ..

[44] Malorni W., Garofalo T., Tinari A., Manganelli V., Misasi R., Sorice M. (2008). Analyzing lipid raft dynamics during cell apoptosis. Methods Enzymol..

[45] Jalmar O., Garcia-Saez A.J., Berland L., Gonzalvez F., Petit P.X. (2010). Giant unilamellar vesicles (GUVs) as a new tool for analysis of caspase-8/Bid-FL complex binding to cardiolipin and its functional activity. Cell Death Dis..

[46] Jalmar O., Francois-Moutal L., Garcia-Saez A.J., Perry M., Granjon T., Gonzalvez F., Gottlieb E., Ayala-Sanmartin J., Klosgen B., Schwille P. (2013). Caspase-8 binding to cardiolipin in giant unilamellar vesicles provides a functional docking platform for bid. PLoS ONE.

[47] Bleicken S., Garcia-Saez A.J., Conte E., Bordignon E. (2012). Dynamic interaction of cBid with detergents, liposomes and mitochondria. PLoS ONE.

[48] Unsay J.D., Cosentino K., Sporbeck K., Garcia-Saez A.J. (2017). Pro-apoptotic cBid and Bax exhibit distinct membrane remodeling activities: An AFM study. Biochim. Biophys. Acta Biomembr..

[49] Schug Z.T., Gonzalvez F., Houtkooper R.H., Vaz F.M., Gottlieb E. (2011). BID is cleaved by caspase-8 within a native complex on the mitochondrial membrane. Cell Death Differ..

[50] Flores-Romero H., Hohorst L., John M., Albert M.C., King L.E., Beckmann L., Szabo T., Hertlein V., Luo X., Villunger A. (2022). BCL-2-family protein tBID can act as a BAX-like effector of apoptosis. EMBO J..

[51] Bleicken S., Landeta O., Landajuela A., Basanez G., Garcia-Saez A.J. (2013). Proapoptotic Bax and Bak form stable protein-permeable pores of tunable size. J Biol Chem..

[52] Hockings C., Anwari K., Ninnis R.L., Brouwer J., O’Hely M., Evangelista M., Hinds M.G., Czabotar P.E., Lee E.F., Fairlie W.D. (2015). Bid chimeras indicate that most BH3-only proteins can directly activate Bak and Bax, and show no preference for Bak versus Bax. Cell Death Dis..

[53] Moldoveanu T., Grace C.R., Llambi F., Nourse A., Fitzgerald P., Gehring K., Kriwacki R.W., Green D.R. (2013). BID-induced structural changes in BAK promote apoptosis. Nat. Struct. Mol. Biol..

[54] Sarosiek K.A., Chi X., Bachman J.A., Sims J.J., Montero J., Patel L., Flanagan A., Andrews D.W., Sorger P., Letai A. (2013). BID preferentially activates BAK while BIM preferentially activates BAX, affecting chemotherapy response. Mol. Cell.

[55] Petit P.X., Dupaigne P., Pariselli F., Gonzalvez F., Etienne F., Rameau C., Bernard S. (2009). Interaction of the alpha-helical H6 peptide from the pro-apoptotic protein tBid with cardiolipin. FEBS J..

[56] Gonzalvez F., Pariselli F., Jalmar O., Dupaigne P., Sureau F., Dellinger M., Hendrickson E.A., Bernard S., Petit P.X. (2010). Mechanistic issues of the interaction of the hairpin-forming domain of tBid with mitochondrial cardiolipin. PLoS ONE.

[57] Cartron P.F., Gallenne T., Bougras G., Gautier F., Manero F., Vusio P., Meflah K., Vallette F.M., Juin P. (2004). The first alpha helix of Bax plays a necessary role in its ligand-induced activation by the BH3-only proteins Bid and PUMA. Mol. Cell.

[58] Juin P., Cartron P.F., Vallette F.M. (2005). Activation of Bax by BH3 domains during apoptosis: The unfolding of a deadly plot. Cell Cycle.

[59] Garcia-Saez A.J., Mingarro I., Perez-Paya E., Salgado J. (2004). Membrane-insertion fragments of Bcl-xL, Bax, and Bid. Biochemistry.

[60] Garcia-Saez A.J., Coraiola M., Dalla Serra M., Mingarro I., Menestrina G., Salgado J. (2005). Peptides derived from apoptotic Bax and Bid reproduce the poration activity of the parent full-length proteins. Biophys. J..

[61] Cartron P.F., Oliver L., Mayat E., Meflah K., Vallette F.M. (2004). Impact of pH on Bax alpha conformation, oligomerisation and mitochondrial integration. FEBS Lett..

[62] Guna A., Stevens T.A., Inglis A.J., Replogle J.M., Esantsi T.K., Muthukumar G., Shaffer K.C.L., Wang M.L., Pogson A.N., Jones J.J. (2022). MTCH2 is a mitochondrial outer membrane protein insertase. Science.

[63] Bartos L., Menon A.K., Vacha R. (2024). Insertases scramble lipids: Molecular simulations of MTCH2. Structure.

[64] Peng X., Yang Y., Hou R., Zhang L., Shen C., Yang X., Luo Z., Yin Z., Cao Y. (2024). MTCH2 in Metabolic Diseases, Neurodegenerative Diseases, Cancers, Embryonic Development and Reproduction. Drug Des. Devel. Ther..

[65] Raemy E., Montessuit S., Pierredon S., van Kampen A.H., Vaz F.M., Martinou J.C. (2016). Cardiolipin or MTCH2 can serve as tBID receptors during apoptosis. Cell Death Differ..

[66] Cimprich K.A., Cortez D. (2008). ATR: An essential regulator of genome integrity. Nat. Rev. Mol. Cell Biol..

[67] Zeman M.K., Cimprich K.A. (2014). Causes and consequences of replication stress. Nat. Cell Biol..

[68] Sancar A., Lindsey-Boltz L.A., Unsal-Kacmaz K., Linn S. (2004). Molecular mechanisms of mammalian DNA repair and the DNA damage checkpoints. Annu. Rev. Biochem..

[69] Bartkova J., Horejsi Z., Koed K., Kramer A., Tort F., Zieger K., Guldberg P., Sehested M., Nesland J.M., Lukas C. (2005). DNA damage response as a candidate anti-cancer barrier in early human tumorigenesis. Nature.

[70] Matsuoka S., Ballif B.A., Smogorzewska A., McDonald E.R., Hurov K.E., Luo J., Bakalarski C.E., Zhao Z., Solimini N., Lerenthal Y. (2007). ATM and ATR substrate analysis reveals extensive protein networks responsive to DNA damage. Science.

[71] Cortez D., Guntuku S., Qin J., Elledge S.J. (2001). ATR and ATRIP: Partners in checkpoint signaling. Science.

[72] Singh P., Ravanan P., Talwar P. (2016). Death Associated Protein Kinase 2016, 1 (DAPK1): A Regulator of Apoptosis and Autophagy. Front. Mol. Neurosci..

[73] Zinkel S.S., Hurov K.E., Ong C., Abtahi F.M., Gross A., Korsmeyer S.J. (2005). A role for proapoptotic BID in the DNA-damage response. Cell.

[74] Maryanovich M., Oberkovitz G., Niv H., Vorobiyov L., Zaltsman Y., Brenner O., Lapidot T., Jung S., Gross A. (2012). The ATM-BID pathway regulates quiescence and survival of haematopoietic stem cells. Nat. Cell Biol..

[75] Tasdogan A., Kumar S., Allies G., Bausinger J., Beckel F., Hofemeister H., Mulaw M., Madan V., Scharffetter-Kochanek K., Feuring-Buske M. (2016). DNA Damage-Induced HSPC Malfunction Depends on ROS Accumulation Downstream of IFN-1 Signaling and Bid Mobilization. Cell Stem Cell.

[76] Cao F., Zhou T., Simpson D., Zhou Y., Boyer J., Chen B., Jin T., Cordeiro-Stone M., Kaufmann W. (2007). p53-Dependent but ATM-independent inhibition of DNA synthesis and G2 arrest in cadmium-treated human fibroblasts. Toxicol. Appl. Pharmacol..

[77] Bar-Lev Y., Moshitch-Moshkovitz S., Tsarfaty G., Kaufman D., Horev J., Resau J.H., Tsarfaty I. (2016). Mimp/Mtch2, an Obesity Susceptibility Gene, Induces Alteration of Fatty Acid Metabolism in Transgenic Mice. PLoS ONE.

[78] Buzaglo-Azriel L., Kuperman Y., Tsoory M., Zaltsman Y., Shachnai L., Zaidman S.L., Bassat E., Michailovici I., Sarver A., Tzahor E. (2016). Loss of Muscle MTCH2 Increases Whole-Body Energy Utilization and Protects from Diet-Induced Obesity. Cell Rep..

[79] Rottiers V., Francisco A., Platov M., Zaltsman Y., Ruggiero A., Lee S.S., Gross A., Libert S. (2017). MTCH2 is a conserved regulator of lipid homeostasis. Obesity.

[80] Bahat A., Goldman A., Zaltsman Y., Khan D.H., Halperin C., Amzallag E., Krupalnik V., Mullokandov M., Silberman A., Erez A. (2018). MTCH2-mediated mitochondrial fusion drives exit from naive pluripotency in embryonic stem cells. Nat. Commun..

[81] Goldman A., Mullokandov M., Zaltsman Y., Regev L., Levin-Zaidman S., Gross A. (2024). MTCH2 cooperates with MFN2 and lysophosphatidic acid synthesis to sustain mitochondrial fusion. EMBO Rep..

[82] Chourasia S., Petucci C., Shoffler C., Abbasian D., Wang H., Han X., Sivan E., Brandis A., Mehlman T., Malitsky S. (2025). MTCH2 controls energy demand and expenditure to fuel anabolism during adipogenesis. EMBO J..

[83] Zhao X.Y., Zhao B.C., Li H.L., Liu Y., Wang B., Li A.Q., Zeng T.S., Hui H.X., Sun J., Cikes D. (2025). MTCH2 Suppresses Thermogenesis by Regulating Autophagy in Adipose Tissue. Adv. Sci..

[84] Ni H.M., Baty C.J., Li N., Ding W.X., Gao W., Li M., Chen X., Ma J., Michalopoulos G.K., Yin X.M. (2010). Bid agonist regulates murine hepatocyte proliferation by controlling endoplasmic reticulum calcium homeostasis. Hepatology.

[85] Yan S., Zhou J., Zhang H., Lin Z., Khambu B., Liu G., Ma M., Chen X., Chalasani N., Yin X.M. (2022). Promotion of diet-induced obesity and metabolic syndromes by BID is associated with gut microbiota. Hepatol. Commun..

[86] Degli Esposti M. (2002). Sequence and functional similarities between pro-apoptotic Bid and plant lipid transfer proteins. Biochim. Biophys. Acta.

[87] Wu C., Wang T., Ghosh A., Long F., Sharma A.K., Dahlby T., Noe F., Severi I., Colleluori G., Cinti S. (2025). MTCH2 modulates CPT1 activity to regulate lipid metabolism of adipocytes. Nat. Commun..

[88] Zhang X., Li E., Kuang Y., Gai Y., Feng Y., Huang Y., Wei Z., Niu J., Yu S., Yang Z. (2025). MTCH2 regulates NRF2-mediated RRM1 expression to promote melanoma proliferation and dacarbazine insensitivity. Cell Death Dis..

[89] Guo B., Zhai D., Cabezas E., Welsh K., Nouraini S., Satterthwait A.C., Reed J.C. (2003). Humanin peptide suppresses apoptosis by interfering with Bax activation. Nature.

[90] Zapala B., Kaczynski L., Kiec-Wilk B., Staszel T., Knapp A., Thoresen G.H., Wybranska I., Dembinska-Kiec A. (2010). Humanins, the neuroprotective and cytoprotective peptides with antiapoptotic and anti-inflammatory properties. Pharmacol. Rep..

[91] Morris D.L., Kastner D.W., Johnson S., Strub M.P., He Y., Bleck C.K.E., Lee D.Y., Tjandra N. (2019). Humanin induces conformational changes in the apoptosis regulator BAX and sequesters it into fibers, preventing mitochondrial outer-membrane permeabilization. J. Biol. Chem..

[92] Morris D.L., Johnson S., Bleck C.K.E., Lee D.Y., Tjandra N. (2020). Humanin selectively prevents the activation of pro-apoptotic protein BID by sequestering it into fibers. J. Biol. Chem..

[93] Sunami T., Caschera F., Morita Y., Toyota T., Nishimura K., Matsuura T., Suzuki H., Hanczyc M.M., Yomo T. (2010). Detection of association and fusion of giant vesicles using a fluorescence-activated cell sorter. Langmuir.

[94] Lacronique V., Mignon A., Fabre M., Viollet B., Rouquet N., Molina T., Porteu A., Henrion A., Bouscary D., Varlet P. (1996). Bcl-2 protects from lethal hepatic apoptosis induced by an anti-Fas antibody in mice. Nat. Med..

[95] de la Coste A., Fabre M., McDonell N., Porteu A., Gilgenkrantz H., Perret C., Kahn A., Mignon A. (1999). Differential protective effects of Bcl-xL and Bcl-2 on apoptotic liver injury in transgenic mice. Am. J. Physiol..

[96] Ciarlo L., Manganelli V., Matarrese P., Garofalo T., Tinari A., Gambardella L., Marconi M., Grasso M., Misasi R., Sorice M. (2012). Raft-like microdomains play a key role in mitochondrial impairment in lymphoid cells from patients with Huntington’s disease. J. Lipid Res..

[97] Garofalo T., Manganelli V., Grasso M., Mattei V., Ferri A., Misasi R., Sorice M. (2015). Role of mitochondrial raft-like microdomains in the regulation of cell apoptosis. Apoptosis.

